# Synthesis of Transesterified Palm Olein-Based Polyol and Rigid Polyurethanes from this Polyol

**DOI:** 10.1007/s11746-015-2592-9

**Published:** 2015-02-03

**Authors:** Mohd Zan Arniza, Seng Soi Hoong, Zainab Idris, Shoot Kian Yeong, Hazimah Abu Hassan, Ahmad Kushairi Din, Yuen May Choo

**Affiliations:** Malaysian Palm Oil Board, 6 Persiaran Instusi, Bandar Baru Bangi, 43000 Kajang, Selangor Malaysia

**Keywords:** Palm oil, Polyol, Transesterification, Epoxidation, Ring opening

## Abstract

Transesterification of palm olein with glycerol can increase the functionality by introducing additional hydroxyl groups to the triglyceride structure, an advantage compared to using palm olein directly as feedstock for producing palm-based polyol. The objective of this study was to synthesize transesterified palm olein-based polyol via a three-step reaction: (1) transesterification of palm olein, (2) epoxidation and (3) epoxide ring opening. Transesterification of palm olein yielded approximately 78 % monoglyceride and has an hydroxyl value of approximately 164 mg KOH g^−1^. The effect of formic acid and hydrogen peroxide concentrations on the epoxidation reaction was studied. The relationships between epoxide ring-opening reaction time and residual oxirane oxygen content and hydroxyl value were monitored. The synthesized transesterified palm olein-based polyol has hydroxyl value between 300 and 330 mg KOH g^−1^ and average molecular weight between 1,000 and 1,100 Da. On the basis of the hydroxyl value and average molecular weight of the polyol, the transesterified palm olein-based polyol is suitable for producing rigid polyurethane foam, which can be designed to exhibit desirable properties. Rigid polyurethane foams were synthesized by substituting a portion of petroleum-based polyol with the transesterified palm olein-based polyol. It was observed that by increasing the amount of transesterified palm olein-based polyol, the core density and compressive strength were reduced but at the same time the insulation properties of the rigid polyurethane foam were improved.

## Introduction

Vegetable oil such as palm oil, soybean oil, rapeseed oil, sunflower oil, peanut oil and cotton oil are renewable sources of oils that can be used as industrial feedstocks to replace products that are derived from non-renewable oils. Palm oil is by far the highest yielding biological source of oil-based hydrocarbons and is significantly more efficient than any of other commercial oil crop. Palm oil has a yield capacity of typically about 4–6 tonnes of oil per hectare per year for the best commercial plantation [1]. In 2013, the crude palm oil yield in Malaysia alone was approximately 19.22 million tonnes and was forecasted to increase to 19.52 million tonnes in 2014 [2]. Furthermore, increasing consumer awareness of their social responsibility towards the environment is increasing the demand for renewable resources and environmentally friendly products. In accordance with this scenario, it is desirable to replace petroleum-based polyols with vegetable oil-based alternatives in the production of polyols and polyurethanes.

Vegetable oil is not reactive under the conditions of polyurethane chemistry because it does not have hydroxyl groups. Therefore, it has to be functionalized with hydroxyl groups so that it can be used as a green raw materials for polyurethane products, as well as plasticizers, stabilizers and additives in lubricants. Palm oil and palm oil derivatives appear to be a good eco-friendly resource for bio-based materials because of their abundance in supply and low cost. One of the common feedstocks is palm olein which consists of 53.4 % unsaturated fatty acids and 46.6 % saturated fatty acids [3]. The major fatty acids are palmitic acid and oleic acid.

Vegetable oil polyols can be obtained by introduction of hydroxyl groups at the positions of double bonds by various methods. Some of the methods are hydroformylation [4], epoxidation followed by epoxide ring opening [5, 6], hydrolysis [7], ozonolysis and hydrogenation [8], as well as microbial conversion [9]. These various synthesis methods produce polyols with different structures which, when converted to polyurethane, impart different properties to the final product. Primary hydroxyl groups in polyols are desirable because they undergo rapid polymerization processes and their compositions greatly influence the physical properties of the polymeric products. The distribution of hydroxyl groups in oils and their position in the fatty acid chains affect the properties of the polyurethane network.

The epoxidation of vegetable oil followed by epoxide ring-opening reaction has been intensively studied as it is the most economical route to produce polyols. However, typical palm-based polyols that were produced through this route usually have hydroxyl values of less than 200 mg KOH g^−1^ [5, 10, 11] that limit their applicability in some polymer formulations such as rigid polyurethane foams. Furthermore, the presence of non-reactive moieties in palm oil, such as saturated fatty acids that only act as plasticizers, which would further reduce the suitability of these polyols as raw materials for rigid polyurethane [8].

Transesterification of palm olein with glycerol is expected to increase the functionality of palm olein by introducing primary hydroxyl groups into the triglyceride structure. This reaction is also known as glycerolysis. Theoretically, at the first stage of the reaction, the products will be a mixture of monoglyceride and diglyceride. Then, the diglyceride will react with another glycerol to produce two monoglycerides. Therefore, one triglyceride will be converted to three monoglycerides [12].

Awang et al. [13] prepared monoglyceride from dihydroxystearic acid by using different catalysts such as *para*-toluenesulfonic acid (*p*-TSA), sodium hydrogen sulphate and sulphuric acid. The reaction was done at 150 °C for 4 h. Jeromin et al. [14] used alkaline catalysts such as sodium hydroxide, potassium hydroxide, lithium hydroxide and calcium hydroxide in the production of monoglyceride from methyl ester. The process was carried out at temperatures ranging from 130  to 160 °C under vacuum between 200 and 400 mbar. The use of alkaline catalysts generated soaps that were separated by an extraction process with water. Acid has to be added to the reaction mixture to deactivate the catalyst and stop the reaction. Tanaka et al. [12] prepared palm oil-based polyol through glycerolysis of palm oil in* tert*-butyl alcohol as solvent. Alkali catalyst was used in the reaction, which yielded approximately 70 % monoglyceride. The monoglyceride obtained was directly used in polyurethane formulations.

**Scheme 1:**
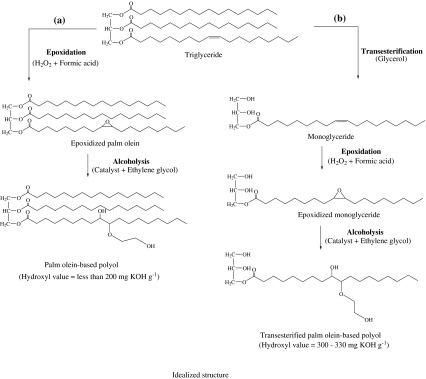
Structural comparison of **a** normal palm olein-based polyol and **b** transesterified palm olein-based polyol

Scheme 1 illustrates the advantages of using transesterified palm olein for producing polyol. Generally, the functionality of polyols increases by addition of new primary hydroxyl groups to the molecule. The monoglyceride produced from glycerolysis of palm olein will provide two additional hydroxyl groups to react with isocyanate to form polyurethane. If using non-transesterified palm olein, the saturated fatty acids stay in the triglyceride structure as non-functional components that do not play a role in the reaction with isocyanate. In the present study, transesterified palm olein-based polyol was prepared through several reaction steps, namely transesterification, epoxidation and ring-opening reaction, and also factors affecting its properties were studied.

## Experimental Procedures

### Materials

Palm olein was supplied by Sime Darby Jomalina Sdn. Bhd. Glycerol was obtained from J. T. Baker. Aluminium hydroxide, sodium carbonate, ethylene glycol, *p*-TSA, bis(trimethylsilyl)trifluoroacetamide and standards for the glycerides were obtained from Sigma Aldrich (M) Sdn. Bhd. Calcium hydroxide, sodium hydroxide and potassium hydroxide were purchased from Fisher Scientific (M) Sdn. Bhd. Formic acid and hydrogen peroxide (50 % purity) were supplied by Kimia Cergas Sdn. Bhd. Sodium chloride and dichloromethane were obtained from Merck Sdn. Bhd. Boron trifluoride–ether complex was obtained from Addit Chemical.

Polyether polyols (Polydo SC 455, hydroxyl value 430–470 mg KOH g^−1^, molecular weight 580 g mol^−1^ with functionality of 4.7 and SC 381, hydroxyl value 365–395 mg KOH g^−1^, molecular weight 764 g mol^−1^ with functionality of 5.2) were supplied by Premium Polyurethane Technology (M) Sdn. Bhd. A polymeric diphenylmethane diisocyanate (Suprasec 5005) was supplied by Huntsman Polyurethanes (UK) Ltd. Its NCO content is 31 % and functionality of 2.7. The cross-linking agent dimethylethanolamine was purchased from Soon Yang Chemicals Sdn. Bhd. Two amine catalysts, Niax A1 and Dabco LV33, were obtained from Kimia Cergas Sdn. Bhd. Dibutyltin dilaurate and Tegostab B 8486 were obtained from Evonik Malaysia Sdn. Bhd. Dibutyltin dilaurate was used as a foaming catalyst. Tegostab B 8486 was used as a surfactant. Distilled water was used as a blowing agent to generate foams. All chemicals were used as received.

### Transesterification of Palm Olein

Palm olein (847 g) was placed in a 2-L glass vessel, equipped with a water cooling condenser, a thermometer, inert gas inlet tube and motorized stirrer. Approximately 553 g of glycerol was added to the glass vessel. Five types of catalyst were investigated: *p*-TSA, calcium hydroxide, aluminium hydroxide, sodium hydroxide or potassium hydroxide at two different concentrations, namely 0.5 and 2 % (w/w) of palm olein. Thus, catalyst was dissolved into the mixture, which was then heated to 150 °C and stirred continuously under dry nitrogen atmosphere. After 6 h, the reaction mixture was transferred to a separation funnel. *n*-Hexane (500 mL) was added to the contents of the separation funnel, which was then shaken vigorously to dissolve the transesterified palm olein (in the *n*-hexane). The mixture was left to separate and the bottom layer, which contained unreacted glycerol, was discarded. The *n*-hexane was removed by evaporation under vacuum. The reaction product (transesterified palm olein) was used to prepare transesterified palm olein-based polyol.

Qualitative analyses of the transesterified palm olein products were carried out by capillary gas chromatography (GC) according to the American Oil Chemists’ Society (AOCS) Official Method Cd 11b-91. The reaction product was treated with bis(trimethylsilyl)trifluoroacetamide in dichloromethane to convert it to trimethylsilyl derivatives. GC analyses of the trimethylsilyl-derivatised samples were carried out by using an HP6890 GC (Agilent Technologies Ltd.) equipped with a flame indicator detector (FID) and a capillary column of HT-5 (cross-linked 5 % phenyl polycarborane-siloxane). This product was obtained with a yield of 85 wt%. FT-IR (cm^−1^): 3,379, 2,920, 2,852 and 1,739.

### Epoxidation

The epoxidation of transesterified palm olein was performed in a 1-L glass vessel, equipped with a thermometer, motorized stirrer, reflux condenser and dropping funnel. The glass vessel was placed in an oil bath set at 60 °C. Transesterified palm olein (500 g) was heated to 60 °C. Performic acid, a premixed reagent of formic acid and 50 wt% hydrogen peroxide, was slowly added dropwise to the transesterified palm olein under fast mechanical stirring. The amounts of formic acid and hydrogen peroxide depend on the expected degree of oxidation of the final products. The oxirane oxygen content was used to monitor the progress of the epoxidation reaction. When oxirane oxygen content of the reaction components reached more than 90 % of the theoretical value, the epoxidation reaction was stopped. The epoxidized transesterified palm olein was then separated from the spent acid and it was neutralized by washing with sodium chloride followed by sodium carbonate until the pH reached between 6 and 7. After that, it was dried under vacuum for several hours until the moisture content was less than 0.05 %. This product was obtained with a yield of 90 wt%. FT-IR (cm^−1^): 3,376, 2,920, 2,852, 1,738, 856 and 835.

### Ring-Opening Reaction

The reactions were carried out in a 500-mL glass vessel, equipped with a thermometer, motorized stirrer, reflux condenser and dropping funnel. A total of 300 g of dried neutralized epoxidized transesterified palm olein was heated to 60 °C. When reaction temperature reached the desired temperature, a mixture of premixed ethylene glycol (21 g) and boron trifluoride–ether complex (1.5 g) was slowly added dropwise to the epoxidized transesterified palm olein under fast mechanical stirring. The reaction was allowed to take place under continuous stirring for between 1 and 3 h. A sample of the reaction mixture was taken every hour to monitor the progress of the reaction through oxirane oxygen content analysis. The reaction was considered completed when the oxirane oxygen content of the epoxidized transesterified palm olein was less than 0.5 %. The polyol obtained was then subjected to neutralization, washing and drying until the moisture content of the polyol was less than 0.05 %. This product was obtained with a yield of 86 wt%. FT-IR (cm^−1^): 3,380, 2,920, 2,850 and 1,736.

### Characterization of Transesterified Palm Olein-Based Polyol

The oxirane oxygen content of epoxidized transesterified palm olein and reaction product was determined following the AOCS Official Method Cd 9-57. The acid value is defined as the amount of potassium hydroxide in milligrams required to neutralize the free acid in 1 g of sample. It was analysed according to AOCS Official Method Te 2a-64. The hydroxyl value and the iodine value were determined according to the AOCS Official Method Cd 13-60 and the AOCS Official Method Cd 1d-92, respectively. The Fourier transform infrared (FT-IR) spectra were recorded in the range of 400–4,000 cm^−1^. The molecular weight distribution was measured using a Varian PL-gel permeation chromatography (GPC) 50 Plus equipped with a differential refractive index (DRI)/viscometer which is a combined detector. The molecular weights of the samples were analysed using a PLgel Mixed D column. Tetrahydrofuran stabilized with 250 ppm butylated hydroxytoluene was used as the eluent and the flow rate was fixed at 1.00 mL min^−1^. The molecular weight distributions were obtained on the basis of a calibration curve generated from polystyrene standards. The polystyrene standards for PLgel Mixed D column used in this study comprised ten vials with molecular weights ranging from 580 to 275,300 Da. For calibration purposes, a concentration of 2 mg mL^−1^ of each of the polystyrene standards was prepared by dissolving it in the tetrahydrofuran before analysis. ^1^H NMR and ^13^C NMR were recorded on 600 MHz Bruker AVANCE III NMR spectrometers with CDCl_3_ as solvent at room temperature.

### Transesterified Palm Olein-Based Polyol

^1^H NMR (CDCl_3_ 600 MHz): *δ* 5.35–5.30 (m, 2H, *H*C=CH), 4.23–4.18 (m, 2H, CH_2_CO–), 4.14–4.10 (m, 2H, C*H*_2_CO–), 3.94–3.88 (m, 2H, C*H*O), 3.83–3.80 (m, 2H, C*H*O), 3.70–3.65 (m, 2H, C*H*_2_O), 3.60–3.50 (m, 2H, C*H*_2_O), 3.40–3.35 (m, 2H, CHO), 2.35–2.30 (m, 2H, OCOC*H*_2_), 2.20–1.70 (m, 2H, C*H*_2_CH=CH), 1.65–1.57 (m, 2H, OCOCH_2_C*H*_2_), 1.36–1.18 (m, 22H, C*H*_2_CH_2_), 0.87 (t, *J* = 7.5 Hz, 3H, C*H*_2_CH_3_). ^13^C NMR (CDCl_3_ 150 MHz): *δ* 178.4 (RO*C*=O), 174.3 (RO*C*=O), 129.7 (*C*H=CH), 74.5 (CHO), 34.1 (O=C*C*H_2_), 31.8–28.9 (*C*H_2_CH_2_), 27.1 (*C*H_2_CH=CH), 24.8 (O=CCH_2_ *C*H_2_), 22.5 (*C*H_2_CH_3_), 13.9 (CH_2_ *C*H_3_).

### Preparation of Polyurethane Rigid Foam

A standard base formulation was used for this part of the experiment. The synthesized transesterified palm olein-based polyol was blended with petrochemical-based polyol in several ratios. The blended polyols, amine catalyst, tin catalyst, surfactant and blowing agent were mixed in a plastic cup using a constant torque homogenizer for several seconds. Then, the required amount of isocyanate was poured into the plastic cup and the mixture was mixed vigorously for 7 s. After that, the mixture was quickly poured into another plastic container (open mould) and foaming reactivity data such as cream time, top of cup, gel time, rise time and tack free time were observed and recorded. Finally, after 5–10 min, the foam was ready to be demoulded from the container. The prepared foam was left for 7 days for aging before cutting it into the required test specimens according to a specific dimension.

### Characterization of Transesterified Palm Olein-Based Polyurethane Rigid Foam

The following physical properties of the polyurethane foam were determined: hardness, core density, thermal conductivity, dimensional stability and compressive strength at 10 % deformation. Hardness was determined with a Shore A durometer according to the method ASTM D 2240-97. Measurements were made at six different points on the sample surface. The average value was taken as the hardness value. Core density was determined according to ASTM D 3574-03, test A. The density of uncored foam was determined by calculation from the mass and volume of the samples. Samples were cut into blocks of 50 mm × 50 mm × 25 mm ± 1 % without skins. The density was calculated in kilograms per cubic metre. Thermal conductivity was determined using a heat flow meter (HFM) from Neztch, Germany according to ASTM C-518, ISO 8301. Samples were placed vertically between two brass plates. Heat is supplied at the top and made to move downwards to stop any convection within the sample. Measurements were taken after the samples had attained equilibrium, about 1–2 h. Dimensional stability of the polyurethane foam was tested at 10, 30, 50 and 70 °C. Samples were cut into cubes of 50 mm × 50 mm × 50 mm without skin. This test consists of exposing foam samples in an air-circulating oven at 120 °C and observing the changes on the properties of foams by naked eye for 5 h. Compressive strength at 10 % deformation was determined according to ISO 844-1978 (E). The test sample, which is cut into blocks of 50 mm × 50 mm × 50 mm without skin, is attached between two rigid plates, fastened in a tensile machine. This test consists of measuring the force necessary to produce a 10 % compression over the entire top area of the foam sample. The test samples must have parallel top and bottom surfaces and vertical sides. The thickness must be no greater than 75 % of the minimum top dimension. The results are calculated in kilopascal. The cell structure of the polyurethane rigid foam was observed using an optical microscope with ×4 magnification. Small pieces of foam were cut off using a razor blade and viewed under the microscope.

## Results and Discussion

The palm olein was characterized prior to the experiments (Table 1). Analysis results show that the palm olein used in this project has an acid value of approximately 5.8 mg KOH g^−1^ and iodine value of 56.5 g I_2_ 100 g^−1^.

**Table 1:** Characteristics of palm olein, its intermediates and transesterified palm olein-based polyol

Parameter	Palm olein	Transesterified palm olein	Epoxidized transesterified palm olein	Transesterified palm olein-based polyol
Moisture (%)	0.1	0.1	0.1	0.1
Acid value (mg KOH g^−1^)	5.8	6.5	6.5	2.0
Iodine value (g I_2_ 100 g^−1^)	56.5	30.7	2.5	2.5
Hydroxyl value (mg KOH g^−1^)	NA	164.0	168.1	300–330
Weight-average molecular weight, Mw (Da)	900–1,000	700–800	800–900	1,000–1,100
Polydispersity index	1.1	1.1	1.1	1.2
Composition (%) (based on percentage area of gas chromatography analysis)				
Fatty acid	0.9	3.6	ND	ND
Monoglyceride	0.3	78.3	ND	ND
Diglyceride	8.3	14.0	ND	ND
Triglyceride	90.5	4.1	ND	ND

*NA* not applicable,* ND* not detected

### Preparation of Transesterified Palm Olein by Glycerolysis

The primary objective of glycerolysis of palm olein is to obtain a high content of monoglyceride in the reaction product. The transesterification of palm olein was conducted according to a method described earlier [13]. Detection of the presence of monoglyceride was done by FT-IR analysis. The presence of a broad peak at 3,379 cm^−1^ in glyceryl monooleate indicated the presence of an hydroxyl group, suggesting that glycerolysis had taken place. Formation of monoglyceride was confirmed by GC analysis. GC results indicated that the reaction product consists of fatty acids, monoglycerides, diglycerides and triglycerides (Table 1).

**Fig. 1:**
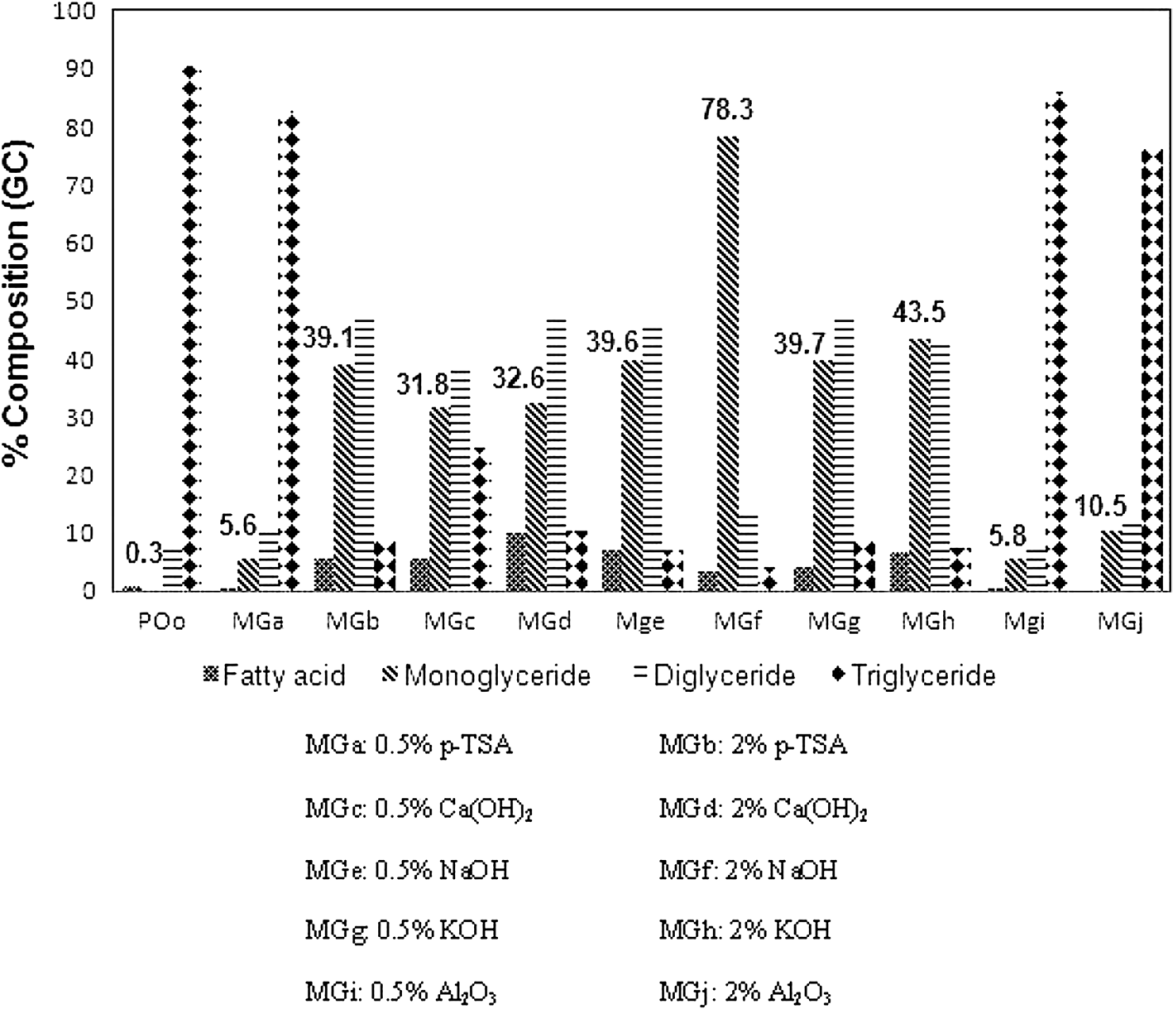
Composition of fatty acid, monoglyceride, diglyceride and triglyceride in transesterified palm olein obtained using various catalysts in transesterification of palm olein with glycerol

Five types of catalysts, i.e. *p*-TSA, calcium hydroxide, aluminium oxide, potassium hydroxide and sodium hydroxide, were used in the transesterification of palm olein with glycerol at 0.5 and 2 % (w/w) of palm olein. The activity of the catalyst in each reaction was evaluated on the basis of the percentage of monoglyceride through GC analysis. Figure 1 shows the percentage composition of transesterified palm olein obtained by using various catalysts in the transesterification of palm olein with glycerol. The composition of palm olein was determined to be 0.9 % fatty acid, 0.3 % monoglyceride, 8.3 % diglyceride and 90.5 % triglyceride. Reaction MGf (2 % NaOH) gave the highest percentage of monoglyceride, 78.3 %, whereas reaction MGa (0.5 %* p*-TSA) gave the lowest, 5.8 %. Therefore, the product from reaction MGf was further used as the starting material for the next reaction, which is epoxidation. The properties of the transesterified palm olein prepared with the optimized reaction parameters are shown in Table 1.

### Preparation of Epoxidized Transesterified Palm Olein

Epoxidation of transesterified palm olein was carried out with performic acid, which was generated from the reaction of hydrogen peroxide and formic acid in acidic medium [15, 16]. Various mole ratios of transesterified palm olein, formic acid and hydrogen peroxide were studied in the epoxidation of transesterified palm olein. Formic acid acts as a catalyst in the formation of the oxirane ring and as a reactant in the hydrolysis of the oxirane ring. The optimum amount of formic acid is therefore crucial to obtain the maximum value of oxirane oxygen content. The aim of this set of studies was to determine the minimum molar ratio of formic acid and hydrogen peroxide per mole of unsaturation of the epoxidized transesterified palm olein that is needed to effectively convert the unsaturation to an epoxide group. The iodine value of the epoxidized transesterified palm olein is a good indication of the effectiveness of the conversion.

**Fig. 2:**
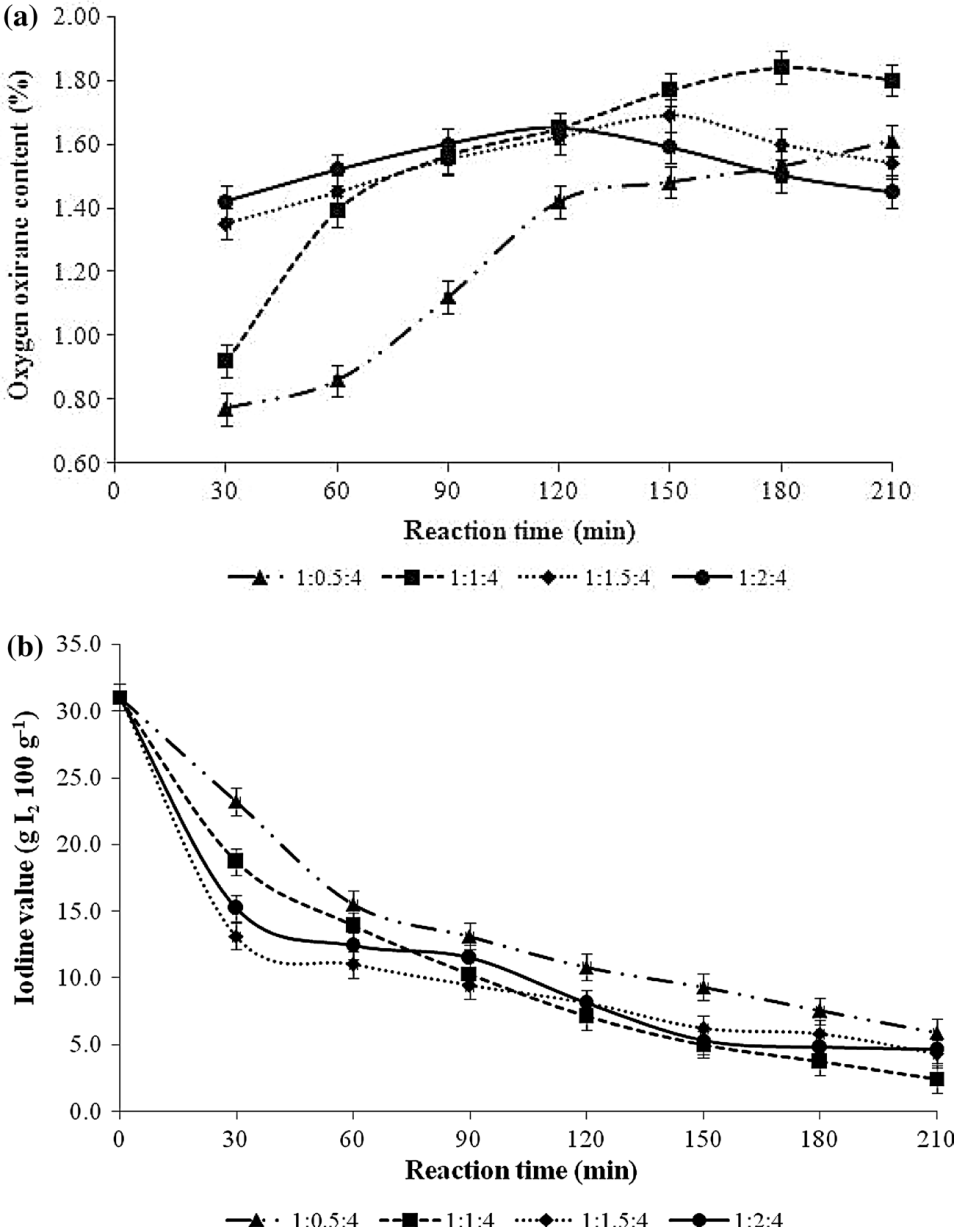
Effect of formic acid concentration on **a** oxirane oxygen content and **b** iodine value of epoxidized transesterified palm olein. Conditions: mole ratio of unsaturation in transesterified palm olein and hydrogen peroxide was fixed at 1 and 4 mol, respectively; mole ratio of formic acid was varied at 0.5, 1, 1.5 and 2 mol; reaction temperature 60 °C; reaction time 3.5 h

The epoxidation process was conducted at a temperature between 50 and 60 °C for 3.5 h. The mole ratio of unsaturation in transesterified palm olein, formic acid and hydrogen peroxide was varied, i.e. 1:0.5:4, 1:1:4, 1:1.5:4 and 1:2:4. Figure 2a shows that the rate of epoxidation increased as the concentration of formic acid in the system increased. A mole ratio of 1:0.5:4 afforded a significant increase of oxirane oxygen content (1.42 %) at 120 min and continued to increase until the end of the reaction. The maximum oxirane oxygen content value (1.84 %) of epoxidized transesterified palm olein was obtained at 180 min for the reaction using a mole ratio of 1:1:4. The percentage conversion was calculated to be 94.8 %. However, increasing the concentration of formic acid to 1.5 and 2 mol did not increase the oxirane oxygen content of epoxidized transesterified palm olein to a value higher than 1.84 %. Figure 2b shows that the iodine values of epoxidized transesterified palm olein for all mole ratios evaluated decreased rapidly in the first hour of reaction. This indicates that almost all the unsaturated carbon–carbon bonds in transesterified palm olein were effectively converted to epoxide groups. The iodine value for a mole ratio of 1:1:4 was the lowest value obtained (2.4 g I_2_ 100 g^−1^) after 210 min of reaction; increasing the concentration of formic acid to 2 mol did not further reduce the iodine value. These results indicated that 1 mol of formic acid is most favourable for effective conversion of the unsaturation groups in transesterified palm olein to epoxide groups.

**Fig. 3:**
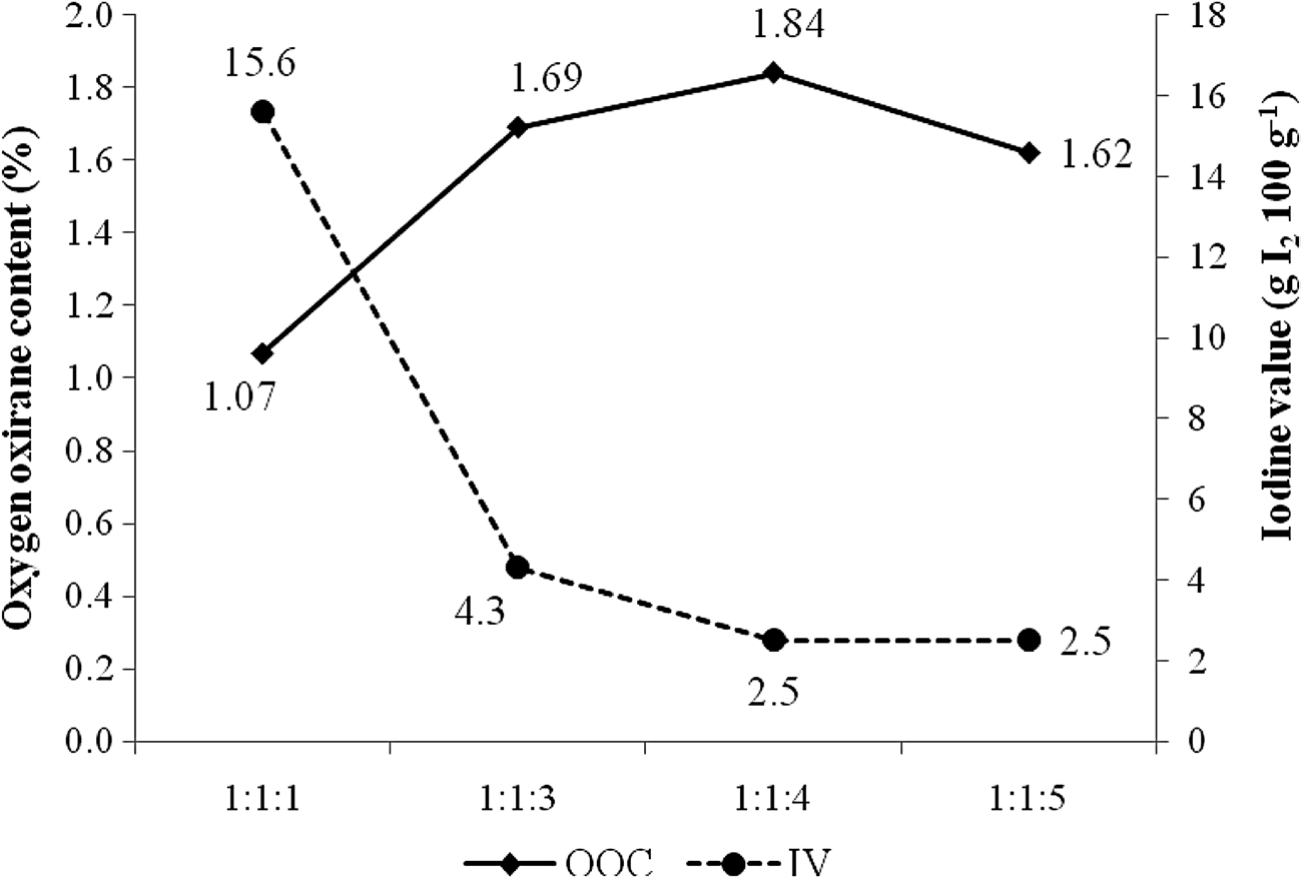
Effect of hydrogen peroxide concentration on oxirane oxygen content (OOC) and iodine value (IV) of epoxidized transesterified palm olein. Conditions: mole ratio of unsaturation in transesterified palm olein and formic acid was fixed at 1 mol; mole ratio of hydrogen peroxide was varied at 1, 3, 4 and 5 mol; reaction temperature 60 °C; reaction time 3.5 h

The effect of hydrogen peroxide concentration was studied by varying the ratio of unsaturation in transesterified palm olein, formic acid and hydrogen peroxide (1:1:1, 1:1:3, 1:1:4 and 1:1:5). Figure 3 shows that the oxirane oxygen content of epoxidized transesterified palm olein increased as the concentration of hydrogen peroxide increased. The highest oxirane oxygen content obtained was 1.84 % by using a mole ratio of 1:1:4 with 94.8 % conversion. Increasing the mole ratio of hydrogen peroxide to 5 mol did not further increase oxirane oxygen content; instead, it reduced the oxirane oxygen content value to 1.62 %. This suggests that 4 mol is the optimum concentration of hydrogen peroxide in the system. The lowest iodine value obtained was 2.5 g I_2_ 100 g^−1^ for both mole ratios of 1:1:4 and 1:1:5, indicating that almost all of the unsaturation of the transesterified palm olein had been converted to epoxide groups.

**Fig. 4:**
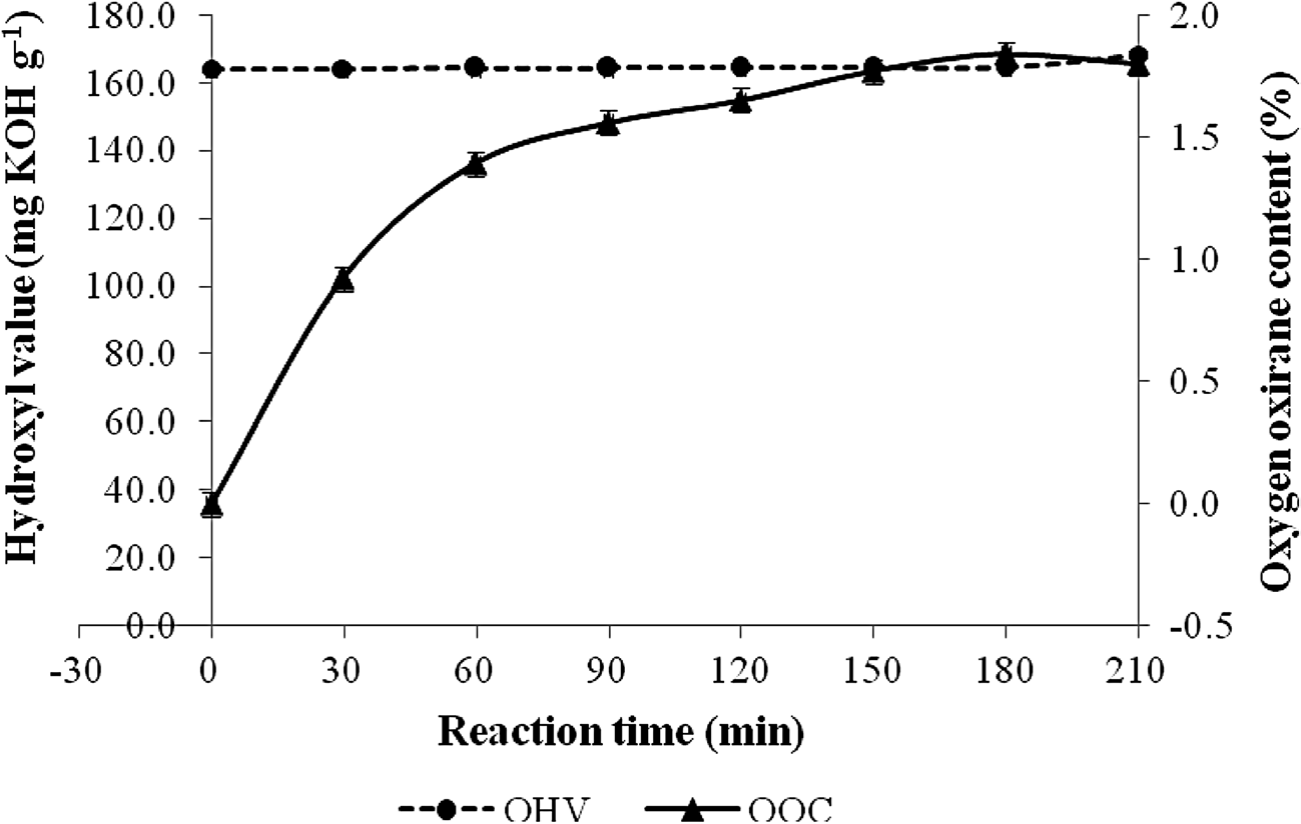
Hydroxyl value (OHV) profile during epoxidation of transesterified palm olein. Conditions: mole ratio of unsaturation in transesterified palm olein/formic acid/hydrogen peroxide (1:1:4), reaction temperature 60 °C, reaction time 3.5 h

The effect of epoxidation on the hydroxyl value of epoxidized transesterified palm olein was also monitored (Fig. 4). The initial hydroxyl value for transesterified palm olein was approximately 164 mg KOH g^−1^. During the first 3 h of the epoxidation reaction, the hydroxyl value was consistent. However, at 210 min of reaction, as the oxirane oxygen content started to reduce, the hydroxyl value of epoxidized transesterified palm olein slightly increased to 168.1 mg KOH g^−1^ sample. This indicates that a side reaction in which the epoxide group was ring opened to a dihydroxy group possibly occurred at the optimum value.

The best reaction conditions for the preparation of epoxidized transesterified palm olein were a mole ratio of transesterified palm olein, formic acid and hydrogen peroxide of 1:1:4, a temperature between 50 and 60 °C and a reaction time of 3 h. The epoxidized transesterified palm olein prepared under these optimized conditions had an oxirane oxygen content value of 1.84 % and iodine value of 2.5 g I_2_ 100 g^−1^. The prepared epoxidized transesterified palm olein was used as the substrate in the alcoholysis to produce polyol.

### Ring Opening of Epoxidized Transesterified Palm Olein

Lewis acid catalyzed ring opening of an epoxide by a polyhydric alcohol generally takes place through a two-step reaction. Firstly, the epoxide is activated by the catalyst, boron trifluoride–ether complex. Then, the polyhydric alcohol will attack the activated epoxide via an S_N_2 mechanism. The polyhydric alcohol used in this study was ethylene glycol as it is the simplest form of diol that is easily available.

**Fig. 5:**
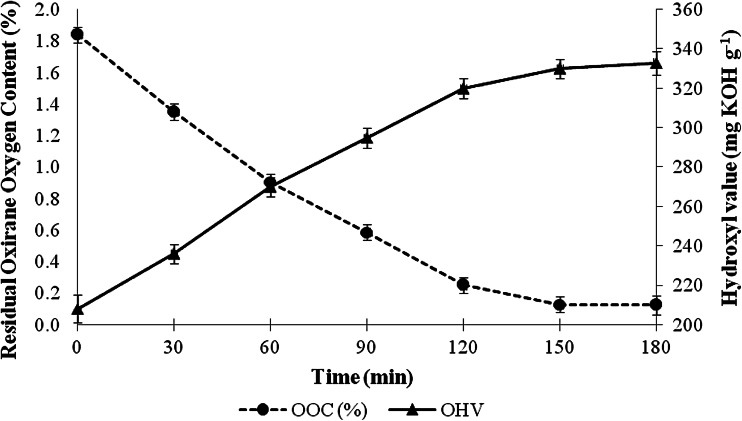
Influence of ring-opening reaction time on the residual oxirane oxygen content and the hydroxyl values of transesterified palm olein-based polyol

The relationships between the ring-opening reaction time and residual oxirane oxygen content and hydroxyl value of the transesterified palm olein-based polyol are given in Fig. 5. As expected, the residual oxirane oxygen content of the product decreased from 1.8 to 0.1 % in 2.5 h of reaction. This indicates that the ring-opening reaction was dependent on the reaction time. The hydroxyl values of transesterified palm olein-based polyols gradually increased with longer ring-opening reaction time, and reached 333 mg KOH g^−1^ at 2.5 h.

FT-IR results show the presence of an hydroxyl absorption at about 3,340 cm^−1^ alongside an ester carbonyl peak at 1,740 cm^−1^, indicating the formation of monoglyceride during transesterification of palm olein. Epoxidized transesterified palm olein was characterized by the appearance of epoxide peaks at 856 and 835 cm^−1^, which suggest that an oxirane ring was successfully formed during epoxidation of transesterified palm olein. Meanwhile, the disappearance of the epoxide peak and the increased intensity of the hydroxyl absorption were indications of a successful ring-opening reaction to produce transesterified palm olein-based polyol.

**Fig. 6:**
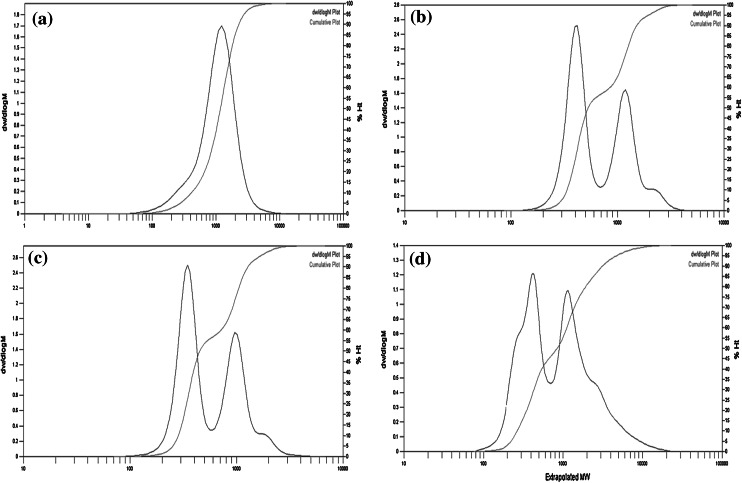
Molecular weight distribution plots for **a** palm olein, **b** transesterified palm olein, **c** epoxidized transesterified palm olein and **d** transesterified palm olein-based polyol

Gel permeation chromatography (GPC) was used to determine the values of the average molecular weight (Mw) of raw materials and the synthesized transesterified palm olein-based polyol (Table 1). The general trend observed for all samples was that the distribution is monomodal for palm olein, but bimodal for transesterified palm olein, epoxidized transesterified palm olein and its polyol (Fig. 6).

Two major peaks were observed on the GPC curve of transesterified palm olein. The peak in the lower molecular weight region (200–600 Da) suggesting formation of low molecular weight molecules such as monoglyceride and diglyceride indicated that transesterification of palm olein and glycerol had occurred. In addition, a shoulder in the higher molecular weight region (2,000–3,000 Da) might be attributed to an oligomer generated by thermal polymerization during transesterification [17]. The GPC curve for epoxidized transesterified palm olein is similar to the curve of transesterified palm olein.

In the GPC curve of transesterified palm olein-based polyol, the shoulder extends further into the higher molecular weight region, indicating that oligomerization side reactions occurred during the ring-opening reactions. Oligomerization occurred when the newly formed hydroxyl reacted with existing epoxy groups [6]. The epoxy groups may also react with hydroxyl groups of monoglycerides and diglycerides in transesterified palm olein.

**Fig. 7:**
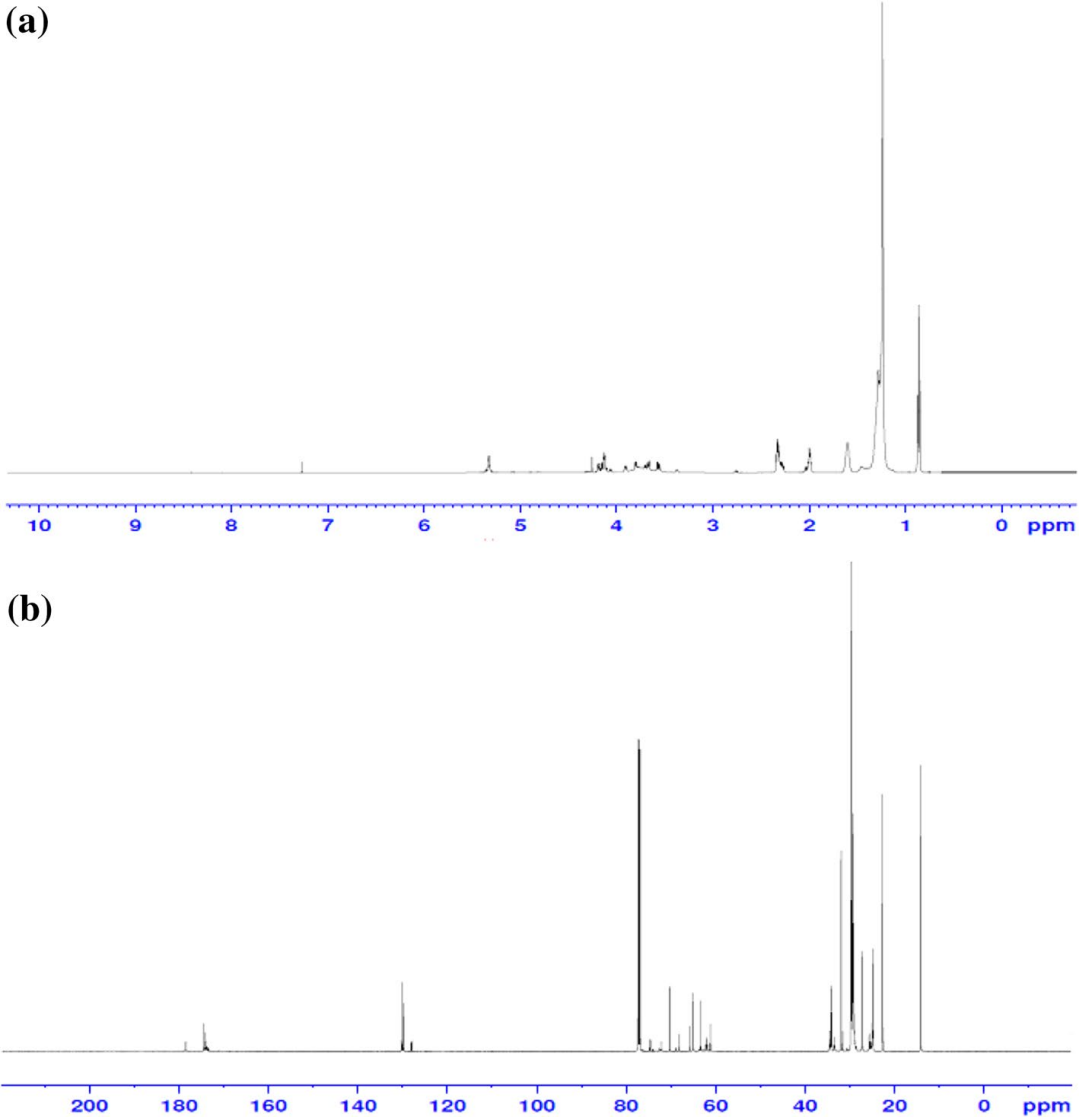
**a** ^1^H and **b** ^13^C NMR of transesterified palm olein-based polyol

Confirmation of the transesterified palm olein-based polyol structure was obtained from the ^1^H and ^13^C NMR spectra (Fig. 7). The characteristic peaks of the transesterified palm olein-based polyol were observed in the *δ* 3.35–3.94 ppm region. The multiple peaks at *δ* 3.35, 3.80 and 3.88 ppm are due to the protons of –C*H*–OH, whereas multiple peaks at *δ* 3.5 and 3.65 ppm are due to the protons of –C*H*_2_–OH. The peaks at *δ* 4.1–4.2 ppm are due to –CH_2_–OH protons of the glycerol moiety, which suggests mono and diglyceride structures. However, the alkene proton signals of –C*H*=CH– at *δ* 5.30–5.35 ppm indicate that the alkene group in the palm olein starting material was not fully converted to an epoxy group during the epoxidation process.

### Potential Application of Transesterified Palm Olein-Based Polyol in Polyurethane (PU)

A preliminary study on using transesterified palm olein-based polyol in PU foam formulation was carried out. A control rigid PU formulation (B1) was used as a benchmark (100 % petroleum-based polyol) and formulation with blends of petroleum polyol and transesterified palm olein-based polyol at ratios of 70:30 (HF1), 60:40 (HF2) and 50:50 (HF3). Reactivity profiles for PU foams using 100 % petroleum-based polyol and blends of petroleum-based polyol and transesterified palm olein-based polyol are shown in Table 2.

**Table 2:** Reactivity profiles for rigid PU foams using 100 % petroleum-based polyol and blends of petroleum-based polyol and transesterified palm olein-based polyol at ratios of 70:30, 60:40 and 50:50, and its properties

	Polyurethane foams			
	B1 (100:0)	HF1 (70:30)	HF2 (60:40)	HF3 (50:50)
Formulation (all amounts in grams)				
Petroleum polyol	100.00	70.00	60.00	50.00
Transesterified palm olein-based polyol	–	30.00	40.00	50.00
Blowing agents	0.15	0.15	0.15	0.15
Chain extender	0.20	0.20	0.20	0.20
Tin catalysts	0.15	0.15	0.15	0.15
Amine catalysts	0.35	0.35	0.35	0.35
Surfactant	1.00	1.00	1.00	1.00
Isocyanate	119.75	111.29	108.48	105.66
Index (NCO/OH)	1.2	1.2	1.2	1.2
Reactivity profile (s)				
Cream time	18	22	30	31
Top of cup	42	75	80	93
Gel time	19	85	94	99
Rise time	37	107	114	120
Tack free time	55	170	208	210
Core density (kg m^−3^)	116.3	94.7	83.8	78.3
Compressive strength (kPa)				
Actual	940.0	798.5	623.0	464.5
Normalized^a^	940.0	765.4	677.3	632.9

B1 is used as the standard rigid PU foam in this work, which is produced by using 100 % petroleum-based polyol

Normalized compressive strength = $\frac{{{\text{actual compressive strengths of benchmark foam }}\left( {\text{B1}} \right)}}{{{\text{core density of benchmark foam }}\left( {\text{B1}} \right)}}$ × core density of the respective foam

^a^The normalized compressive strength value was calculated from actual compressive strength and its respective core density

The incorporation of transesterified palm olein-based polyol into the rigid PU decreased the reactivity of the foaming profile of rigid PU as shown by the increase in cream time, top of cup, gel time, rise time and tack free time as compared to the foaming profile of foam made with 100 % petroleum-based polyol. This occurrence is assumed to be caused by the presence of a secondary hydroxyl group and a palmitoyl moiety in the transesterified palm olein. Incorporation of transesterified palm olein-based polyol produced a yellowish foam compared to the white foam from 100 % petroleum-based polyol. The yellowish appearance was due in part to transesterified palm olein-based polyol which was brownish orange in colour.

Benchmark foam (B1) with a core density of 116.3 kg m^−3^ showed the highest density as 100 % petroleum-based polyol was used in its formulation which had the highest hydroxyl value (Table 2). However, incorporating transesterified palm olein-based polyol in the formulation which had a lower hydroxyl value than the petroleum-based polyol will subsequently reduce the number of hydroxyl groups that are involved in the cross-linking reaction with isocyanate. As a result, the density of HF1 (94.7 kg m^−3^), HF2 (83.8 kg m^−3^) and HF3 (78.3 kg m^−3^) reduced with increasing amount of transesterified palm olein-based polyol in the formulation. Rigid PU foam comprising blends of petroleum-based polyol and transesterified palm olein-based polyol can be applied depending on the application, e.g. pipe insulation required foams having a minimum density of 60 kg m^−3^ [18], while the density of rigid PU foam for decorative and ornamental application has been reported in the range of 30–100 kg m^−3^ [19–21].

Table 2 also shows the compressive strength for all four foams formulated with 100 % petroleum-based polyol and blends of petroleum-based polyol and transesterified palm olein-based polyol. The normalized compressive strength of benchmark foam (B1) is 940.0 kPa, while the values for HF1 (70:30), HF2 (60:40) and HF3 (50:50) foams are 765.4, 677.3 and 632.9 kPa, respectively. The compressive strengths of the rigid PU foams decreased with increasing amount of transesterified palm olein-based polyol in the formulation.

The thermal conductivity of PU foam is inversely proportional to the insulation properties. According to European Standard EN 253, polyurethane foam targeted for pre-insulated pipes must possess a thermal conductivity value lower than 0.033 W mK^−1^ at 50 °C [18]. All four rigid polyurethane foams produced using this formulation have thermal conductivity slightly higher than the standard requirement (Table 3). However, when comparing thermal conductivity between the four foams, incorporation of higher amounts of transesterified palm olein-based polyol in the polyol blends reduced the thermal conductivity value thereby indicating better insulation properties of the foam.

**Table 3:** Thermal conductivity value of rigid PU foams determined at several temperatures

Thermal conductivity (W mK^−1^) at different temperature (°C)	B1 (100:0)	HF1 (70:30)	HF2 (60:40)	HF3 (50:50)
10	0.0374	0.0369	0.0366	0.0362
30	0.0393	0.0390	0.0388	0.0382
50	0.0418	0.0412	0.0404	0.0401
70	0.0432	0.0431	0.0430	0.0429

**Fig. 8:**
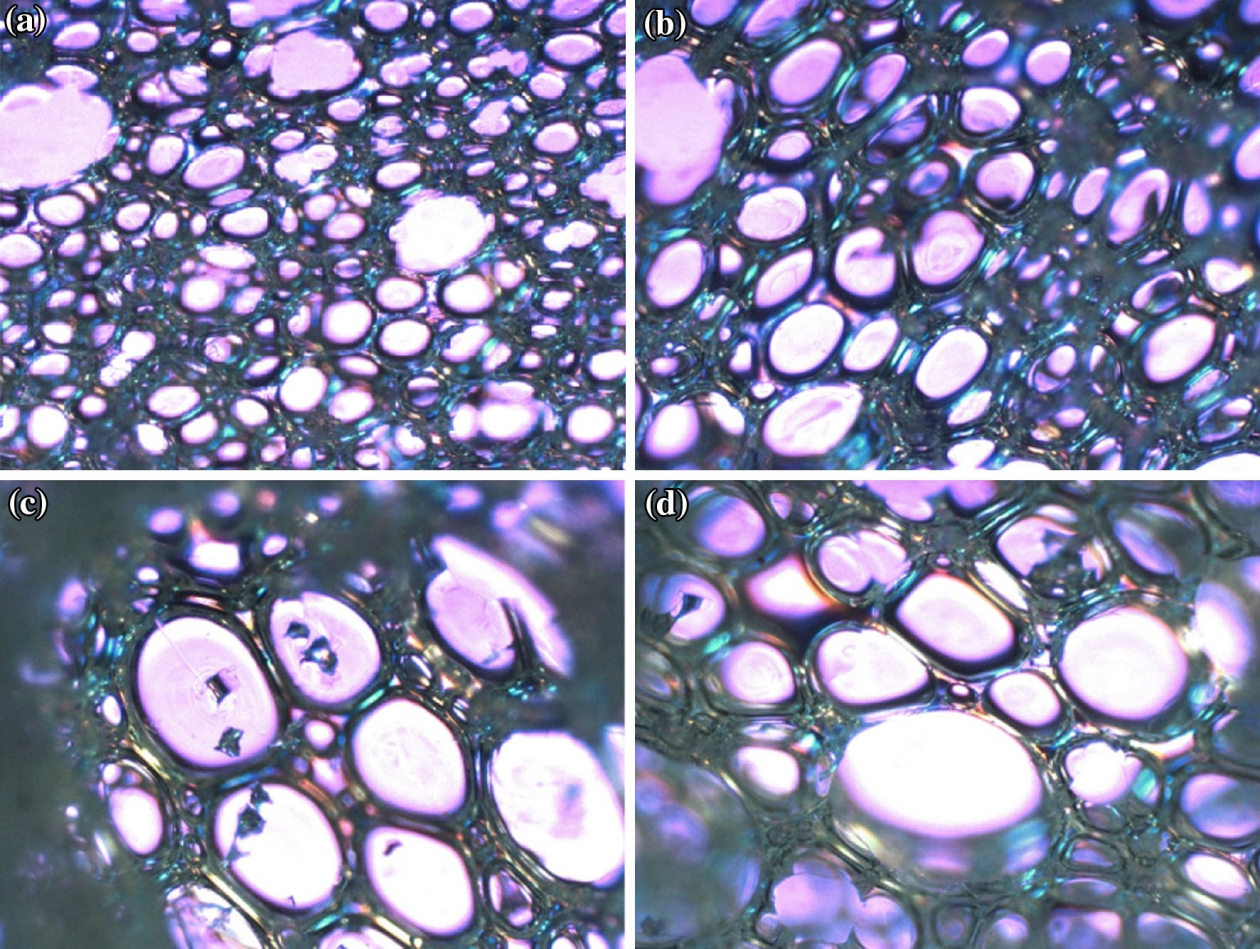
Optical micrographs of cross-sectioned rigid polyurethane foams (×4 magnification): **a** B1 (100:0), **b** HF1 (70:30), **c** HF2 (60:40) and **d** HF3 (50:50)

A series of pictures showing the cell structure of rigid foams were obtained by using an optical microscope (Fig. 8). The cells of all foams showed similar spherical appearance in microstructure. The benchmark foam showed the smallest and most homogenous cell structure owing to the use of 100 % petroleum-based polyol and results in the highest density and compressive strength. However, partial substitution of the petroleum polyol with transesterified palm olein-based polyol increased the size of cells and therefore reduced the density and compressive strength of the foams.

## Conclusion

Transesterified palm olein-based polyol was successfully prepared through a three-step synthesis pathway. In the first step, monoglycerides were derived from palm olein through glycerolysis with a yield between 70 and 80 %. The reaction was catalyzed by sodium hydroxide, which produced monoglycerides with an hydroxyl value of 164 mg KOH g^−1^. The transesterified palm olein was then epoxidized with performic acid; the highest oxygen oxirane content obtained was 1.84 % with approximately 94.8 % conversion of the unsaturated bonds in transesterified palm olein to epoxide groups. The epoxidation does not significantly affect the hydroxyl value of the epoxidized transesterified palm olein. The prepared epoxidized transesterified palm olein was then used as the substrate in the alcoholysis to produce polyol. The final hydroxyl value of the transesterified palm olein-based polyol was approximately 330 mg KOH g^−1^ sample. Incorporation of transesterified palm olein-based polyol in rigid PU formulation resulted in medium density rigid PU foam with better insulation properties compared to standard rigid PU foam.
